# Influence of Hepatitis C Coinfection and Treatment on Risk of Diabetes Mellitus in HIV-Positive Persons

**DOI:** 10.1093/ofid/ofaa470

**Published:** 2020-10-07

**Authors:** Amanda Mocroft, Jens D Lundgren, Juergen K Rockstroh, Inka Aho, Gilles Wandeler, Lars Nielsen, Simon Edwards, Jean-Paul Viard, Karine Lacombe, Gerd Fätkenheuer, Giovanni Guaraldi, Montserrat Laguno, Josep Llibre, Hila Elinav, Leo Flamholc, Martin Gisinger, Dzmitry Paduta, Irina Khromova, David Jilich, Blazej Rozplochowski, Cristiana Oprea, Lars Peters, EuroSIDA study, A Harxhi, M Losso, M Kundro, B Schmied, R Zangerle, I Karpov, A Vassilenko, D Paduto, N Clumeck, S De Wit, M Delforge, E Florence, L Vandekerckhove, V Hadziosmanovic, J Begovac, L Machala, D Jilich, D Sedlacek, G Kronborg, T Benfield, J Gerstoft, T Katzenstein, C Pedersen, I S Johansen, L Ostergaard, L Wiese, N F Moller, K Zilmer, I Aho, J-P Viard, P-M Girard, C Pradier, E Fontas, C Duvivier, J Rockstroh, G Behrens, O Degen, H J Stellbrink, J Bogner, G Fätkenheuer, N Chkhartishvili, H Sambatakou, G Adamis, N Paissios, J Szlávik, M Gottfredsson, C Kelly, L Tau, D Turner, M Burke, E Shahar, G Hassoun, H Elinav, M Haouzi, D Elbirt, A D’arminio Monforte, R Esposito, I Mazeu, C Mussini, F Mazzotta, A Gabbuti, A Lazzarin, A Castagna, N Gianotti, M Galli, A Ridolfo, V Uzdaviniene, R Matulionyte, T Staub, R Hemmer, S Dragas, M Stevanovic, P Reiss, J Trajanovska, D H Reikvam, A Maeland, J Bruun, B Knysz, J Gasiorowski, M Inglot, E Bakowska, R Flisiak, A Grzeszczuk, M Parczewski, K Maciejewska, B Aksak-Was, M Beniowski, E Mularska, E Jablonowska, J Kamerys, K Wojcik, I Mozer-Lisewska, B Rozplochowski, A Zagalo, K Mansinho, F Maltez, C Oprea, A Yakovlev, I Khromova, E Kuzovatova, E Borodulina, E Vdoushkina, J Ranin, J Tomazic, J M Miro, M Laguno, E Martinez, F Garcia, J L Blanco, M Martinez-Rebollar, J Mallolas, P Callau, J Rojas, A Inciarta, S Moreno, B Clotet, A Jou, R Paredes, J Puig, J M Llibre, J R Santos, P Domingo, M Gutierrez, G Mateo, M A Sambeat, J M Laporte, K Falconer, A Thalme, A Sonnerborg, C J Treutiger, L Flamholc, A Scherrer, R Weber, M Cavassini, A Calmy, H Furrer, M Battegay, P Schmid, A Kuznetsova, J Mikhalik, M Sluzhynska, A Milinkovic, A M Johnson, E Simons, S Edwards, A Phillips, M A Johnson, A Mocroft, A Winston, A Clarke, C Leen, I Karpov, M Losso, J Lundgren, J Rockstroh, I Aho, L D Rasmussen, V Svedhem, G Wandeler, C Pradier, N Chkhartishvili, R Matulionyte, C Oprea, J D Kowalska, J Begovac, J M Miró, G Guaraldi, R Paredes, G Wandeler, R Paredes, O Kirk, L Peters, A Bojesen, D Raben, E V Hansen, D Kristensen, J F Larsen, A H Fischer, A Mocroft, A Phillips, A Cozzi-Lepri, S Amele, A Pelchen-Matthews, A Roen

**Affiliations:** 1 Centre for Clinical Research, Epidemiology, Modelling and Evaluation (CREME), Institute for Global Health, University College London, London, UK; 2 CHIP, Rigshospitalet, Copenhagen, Denmark; 3 University Hospital Bonn, Bonn, Germany; 4 Helsinki University Hospital, Helsinki, Finland; 5 Department of Infectious Diseases, Bern University Hospital, University of Bern, Bern, Switzerland; 6 Nordsjællands Hospital, Hillerød, Denmark; 7 Mortimer Market Centre, London, UK; 8 Hôtel-Dieu, AP-HP, Paris, France; 9 Sorbonne Université, IPLESP Inserm UMR-S1136, AP-HP, Paris, France; 10 University Hospital of Cologne, Cologne, Germany; 11 University of Modena and Reggio Emilia, Modena, Italy; 12 Hospital Clinic, Barcelona, Spain; 13 University Hospital Germans Trias i Pujol, Badalona, Barcelona, Spain; 14 Hadassah Hospital, Jerusalem, Israel; 15 Skane University Hospital, Malmö, Sweden; 16 Medical University of Innsbruck, Innsbruck, Austria; 17 Gomel Regional Centre for Hygiene, Gomel, Belarus; 18 Centre for HIV/AIDS & Infectious Diseases, Kaliningrad, Russia; 19 Charles University in Prague and Na Bulovce Hospital, Prague, Czech Republic; 20 Poznan University of Medical Sciences, Poznan, Poland; 21 Carol Davila University of Medicine and Pharmacy, Bucharest, Romania

**Keywords:** diabetes mellitus, direct-acting antivirals, hepatitis C, HIV, sustained virologic response

## Abstract

**Background:**

The role of hepatitis C virus (HCV) coinfection and HCV-RNA in the development of diabetes mellitus (DM) in HIV-positive persons remains unclear.

**Methods:**

Poisson regression was used to compare incidence rates of DM (blood glucose >11.1 mmol/L, HbA1C >6.5% or >48 mmol/mol, starting antidiabetic medicine or physician reported date of DM onset) between current HIV/HCV groups (anti-HCV-negative, spontaneously cleared HCV, chronic untreated HCV, successfully treated HCV, HCV-RNA-positive after HCV treatment).

**Results:**

A total of 16 099 persons were included; at baseline 10 091 (62.7%) were HCV-Ab-negative, 722 (4.5%) were spontaneous clearers, 3614 (22.4%) were chronically infected, 912 (5.7%) had been successfully treated, and 760 (4.7%) were HCV-RNA-positive after treatment. During 136 084 person-years of follow-up (PYFU; median [interquartile range], 6.9 [3.6–13.2]), 1108 (6.9%) developed DM (crude incidence rate, 8.1/1000 PYFU; 95% CI, 7.7–8.6). After adjustment, there was no difference between the 5 HCV strata in incidence of DM (global *P* = .33). Hypertension (22.2%; 95% CI, 17.5%–26.2%) and body mass index >25 (22.0%; 95% CI, 10.4%–29.7%) had the largest population-attributable fractions for DM.

**Conclusions:**

HCV coinfection and HCV cure were not associated with DM in this large study. The biggest modifiable risk factors were hypertension and obesity, and continued efforts to manage such comorbidities should be prioritized.

## Teaser

Cirrhosis, but not HCV coinfection per se was associated with increased incidence of diabetes in a European cohort of >16000 PLWH. The biggest modifiable risk factors for diabetes were hypertension and obesity. Management of comorbidities in PLWH should be prioritised.

Hepatitis C (HCV) monoinfection has been associated with an increased risk of a wide range of extrahepatic comorbidities [1], including cardiovascular disease and chronic kidney disease [2, 3], and has also been associated with higher mortality from cardiovascular disease, cancer, and renal disease [4, 5]. Most studies have reported an increased risk of diabetes mellitus (DM) in HCV-positive vs HCV-negative individuals, with a recent meta-analysis reporting a 1.6-fold increased odds [6]. However, a large French cohort study among HIV/HCV-coinfected individuals found that DM was associated with cirrhosis but not HCV infection per se [7], while a large US study of HCV-monoinfected persons found that DM was associated with elevated ALT and gamma-GT, but not HCV itself [8]. The introduction of direct-acting antivirals (DAAs) for the treatment of HCV has had a major impact on HCV treatment [9], with cure rates in excess of 90% in persons coinfected with both HIV and HCV [10]. Data from the pre-DAA era of interferon-based HCV therapy have indicated that sustained virologic response (SVR) could also have indirect beneficial effects in terms of improvements in lifestyle factors, a so-called epiphany effect [11], which could also potentially impact the risk for diabetes. In studies of persons coinfected with HCV and HIV, persons with SVR had a significantly lower incidence of DM compared with those treated for HCV without SVR [12]. A lower incidence of DM among HCV treatment responders vs nonresponders was also found in a Spanish cohort of HIV/HCV-coinfected persons [13]. Both previous studies were based on relatively few cases of DM and were conducted before the introduction of direct-acting antivirals, and therefore larger studies with substantial follow-up and well-defined end points are required to further understand the role of HCV on the development of DM in HIV-positive persons.

The aim of this study was therefore to investigate the incidence of DM in a large pan-European cohort study according to HCV status in HIV-coinfected persons across 5 strata: anti-HCV-negative individuals, spontaneous HCV-RNA clearers, those with chronic untreated HCV infection, those with cured HCV, or those who have been treated but are HCV-RNA-positive.

## METHODS

### The EuroSIDA Study

Persons were included from the EuroSIDA study, a large prospective observational cohort of almost 23 000 HIV-1-positive patients followed in 100 hospitals in 35 European countries plus Israel and Argentina. Individuals were enrolled into 10 cohorts from 1994 onward. In cohort 10, all HIV-positive patients were also required to be positive for anti-HCV antibodies (HCV-RNA-positive, -negative, or unknown status). At recruitment, in addition to demographic and clinical data, a complete antiretroviral therapy history was obtained, together with the most recent CD4 cell counts and HIV-RNA measurements, as well as all HCV tests, HCV-RNA, HCV genotype, hepatitis B surface antigen (HBsAg), and hepatitis B virus (HBV) DNA. Data are collected prospectively at clinical sites and sent to the coordinating center at yearly intervals. At each follow-up visit, all CD4 cell counts, HIV-RNA, HCV tests, HCV-RNA, genotype, and HBsAg results measured since last follow-up are collected, together with start and stop dates for antiretroviral drugs and HCV and HBV drugs. Detailed information about data collected in EuroSIDA can be found at http://www.chip.dk/Ongoing-Studies/EuroSIDA/About.

### Patient Consent Statement

Informed patient consent was obtained according to local and/or national ethics committee requirements; consent was obtained from each particpant before any study-related procedure was performed and in accordance with the International Conference on Harmonisation of Technical Requirements for Registration of Pharmaceuticals for Human Use (ICH) – Good Clinical Practice Guidelines. Further information is available at https://www.chip.dk/Portals/0/files/Eurosida/EuroSIDA/EuroSIDA_Protocol_v4_2019JULI05.pdf?ver=2019-10-02-145631-730 (ClinicalTrials.gov Identifier: NCT02699736).

### Definitions

DM was defined according to laboratory values (blood glucose levels >11.1 mmol/L or HbA1C >6.5%/48 mmol/L) and/or use of antidiabetic medication. We used a blood glucose >11.1 mmol/L to be conservative, reflecting missing information on whether glucose was measured in a fasting state or not. According to the EuroSIDA Manual of Operations, DM can be defined by sending the laboratory data, by the use of antidiabetic medication, or the site can report the clinical diagnosis based on these criteria without sending laboratory data (https://www.chip.dk/Portals/0/files/RESPOND/RESPOND%20Manual%20of%20Operations%20MOOP__Version%201.6.pdf?ver=2019-11-05-124535-643). Baseline was defined as the earliest date after cohort enrollment or January 1, 2001 (when collection of prospective information on DM began), with known HCV serostatus and, for those who were anti-HCV-positive, known HCV-RNA status. Persons aged <18 years at baseline or without a CD4 count and HIV viral load in the 12 months before or 1 month after baseline were excluded, as were persons with DM before baseline.

Based on time-updated HCV antibody tests, HCV-RNA, and HCV treatment, we defined 5 HCV groups, as previously published [14]:

1. anti-HCV-negative;
2. HCV antibody–positive, HCV-RNA-negative, untreated (spontaneous clearers);
3. HCV antibody–positive, HCV-RNA-positive, untreated (chronic infections);
4. HCV antibody–positive, HCV-RNA-negative, treated (successfully treated with any HCV therapy; cured);
5. HCV antibody–positive, HCV-RNA-positive, treated (treated, HCV-RNA positive).

These 5 strata were defined on the basis of latest anti-HCV test and HCV-RNA measurement. Those treated and HCV-RNA-positive included persons who did not achieve SVR, persons who were HCV-RNA-positive who had started treatment more recently, those reinfected with HCV, and persons without an HCV-RNA measurement after treatment completion and lacking an end-of-treatment response. Persons were followed until their last visit (median June 2018), date of death, or DM, whichever occurred first. Person-years of follow-up (PYFU) and DM events accrued according to current HCV stratum using the last observation carried forward, and persons could contribute PYFU to >1 stratum.

### Statistical Analysis

Characteristics of patients were compared across strata using chi-square tests for categorical variables and the Kruskal-Wallis test for continuous data. Incidence rates of DM per 1000 PYFU were calculated within HCV groups, and Poisson regression was used to compare these rates with persons cured as the reference group. In addition to univariate analyses, we adjusted for all factors fixed at baseline (except HCV status) and all factors updated. Factors included were gender, HIV exposure group, region of Europe (North, Central West, South, Central East, East, and Argentina) [15], development of chronic kidney disease [14], HIV viral load, prior AIDS, cumulative exposure to zidovudine, stavudine, and didanosine [16], cardiovascular disease, non-AIDS-defining malignancies (NADMs), end-stage liver disease (ESLD; ascites, hepatorenal syndrome, grade III/IV hepatic encephalopathy, unspecified liver decompensation, esophageal variceal bleeding, spontaneous bacterial peritonitis, liver transplantation, and hepatocellular carcinoma; further information about these events is available at https://www.chip.dk/Studies/EuroSIDA/Study-documents), smoking status (never smoked, current smoker, past smoker, unknown smoking status), hypertension [17], body mass index, CD4, nadir CD4, age, and baseline date. Liver fibrosis was defined according to our previous study [18]. In brief, data were available on all liver biopsy and Fibroscan test results performed at participating centers. Information on aspartate transaminase (AST) and platelet counts was used to calculate the AST-to-platelet ratio (APRI). Hyaluronic acid was available for a small subset. The most recent fibrosis marker measured (before baseline or time-updated) was used to define fibrosis stage, and where >1 marker was measured, priority was given to biopsy, Fibroscan, APRI, and hyaluronic acid. A priori, we investigated the interaction between age and HCV strata.

We performed a wide range of sensitivity analyses to investigate the robustness of our results to different assumptions. These included carrying the last HCV-RNA measurement forward for a maximum of 12 months, excluding persons with stage F3/F4 liver fibrosis at baseline, excluding men who have sex with men (MSM) with a more recent HCV infection, and an analysis limited to post-2014, when DAAs became more widely available for persons included in the EuroSIDA study [19]. In addition, we investigated excluding centers in EuroSIDA where the 95% CI for the center did not include the population point estimate, as incidence of DM could depend on frequency of laboratory testing within centers.

To address which risk factors have the strongest impact on the development of DM, we calculated the population-attributable risk fraction (PAF) for the key identified risk factors. PAF expresses the proportion of events that could have been avoided had that risk factor not been present and considers both the strengths of the associations and the incidence rate of the risk factor.

All analyses were performed in SAS, version 9.4 (SAS Institute, Cary, NC, USA).

## RESULTS

Of 22 828 persons enrolled in the EuroSIDA study, 3948 were excluded as their HCV status and/or HCV-RNA status was unknown, 754 were excluded with DM before the study baseline, 1439 were excluded with no prospective follow-up, and 588 were excluded with missing CD4 and/or HIV viral load at baseline. Compared with the 16 099 included in these analyses, the 2029 excluded due to no follow-up or missing CD4/viral loads were more likely to be females, be younger, have a prior AIDS diagnosis, have fibrosis stage F3/F4 at baseline, be from Central Eastern Europe, have an earlier baseline date, and be HBsAg-positive.

Characteristics of the 16 099 included individuals are shown in Table 1, stratified by HCV strata at baseline. The majority of those included were in group 1 at baseline (HCV-Ab negative; 10 091; 62.7%), followed by those in group 3 (chronically infected; n = 3614; 22.4%), with smaller numbers in group 2 (spontaneous clearers; n = 722; 4.5%), group 4 (successfully treated; n = 912; 5.7%), and group 5 (treated, HCV-RNA positive; n = 760; 4.7%). The HCV strata were heterogeneous at baseline, with a lower burden of fibrosis and of comorbidities in group 1 (HCV-Ab negative) compared with other groups. The median baseline age (interquartile range [IQR]) was 41 (35–49) years, the CD4 count (IQR) was 443/mm^3^ (288–633/mm^3^), and the baseline date (IQR) was January 2006 (January 2001–May 2012).

**Table 1.** Characteristics at Baseline

						HCV Antibody–Positive							
				Anti-HCV-Negative		Group 2		Group 3		Group 4		Group 5	
		All		Group 1		Spontaneous Clearers		Chronic Untreated Infection		Successfully Treated		Treated; HCV-RNA-Positive	
		No.	%	No.	%	No.	%	No.	%	No.	%	No.	%
All		16 099	100.0	10 091	62.7	722	4.5	3614	22.4	912	5.7	760	4.7
Gender	Male	11 910	74.0	7649	75.8	472	65.4	2548	70.5	679	74.5	562	73.9
	Female	4189	26.0	2442	24.2	250	34.6	1066	29.5	233	25.5	198	26.1
HIV risk	MSM	6248	38.8	5306	52.6	99	13.7	427	11.8	250	27.4	166	21.8
	IDU	4026	25.0	293	2.9	411	56.9	2422	67.0	467	51.2	433	57.0
	Heterosexual	4628	28.7	3770	37.4	134	18.6	498	13.8	124	13.6	102	13.4
	Other	1197	7.4	722	7.2	78	10.8	267	7.4	71	7.8	59	7.8
Ethnic	White	13 743	85.4	8483	84.1	589	81.6	3291	91.1	728	79.8	652	85.8
Origin	Other	2356	14.6	1608	15.9	133	18.4	323	8.9	184	20.2	108	14.2
Region	South	3936	24.4	2220	22.0	147	20.4	1015	28.1	259	28.4	295	38.8
	Central	4336	26.9	2858	28.3	238	33.0	692	19.1	331	36.3	217	28.6
	North	3451	21.4	2493	24.7	131	18.1	591	16.4	134	14.7	102	13.4
	Central East	2132	13.2	1328	13.2	89	12.3	602	16.7	53	5.8	60	7.9
	East	1728	10.7	763	7.6	107	14.8	652	18.0	128	14.0	78	10.3
	Argentina	516	3.2	429	4.3	10	1.4	62	1.7	7	0.8	8	1.1
HBV status	Negative	13 555	84.2	8830	87.5	526	72.9	2834	78.4	757	83.0	608	80.0
	Positive	1214	7.5	744	7.4	114	15.8	254	7.0	61	6.7	41	5.4
	Unknown	1330	8.3	517	5.1	82	11.4	526	14.6	94	10.3	111	14.6
Ever cART	No	2395	14.9	1627	16.1	80	11.1	524	14.5	88	9.6	76	10.0
	Yes	13 704	85.1	8464	83.9	642	88.9	3090	85.5	824	90.4	684	90.0
HIV VL	<500	11 036	68.6	6623	65.6	542	75.1	2431	67.3	795	87.2	645	84.9
Copies/mL	>500	5063	31.4	3468	34.4	180	24.9	1183	32.7	117	12.8	115	15.1
Comorbidities	AIDS	4089	25.4	2735	27.1	199	27.6	867	24.0	139	15.2	149	19.6
	CVD	307	1.9	198	2.0	11	1.5	53	1.5	30	3.3	15	2.0
	NADM	311	1.9	181	1.8	20	2.8	73	2.0	21	2.3	16	2.1
	ESLD	175	1.1	45	0.4	10	1.4	66	1.8	23	2.5	31	4.1
	Hypertension	3443	21.4	2233	22.1	161	22.3	635	17.6	229	25.1	185	24.3
	CKD	108	0.7	34	0.3	9	1.2	32	0.9	23	2.5	10	1.3
		All		Anti-HCV Negative		HCV Antibody Positive							
				Group 1		Group 2		Group 3		Group 4		Group 5	
						Spontaneous Clearers		Chronic Untreated Infection		Cured		Treated; HCV-RNA-Positive	
		No.	%	No.	%	No.	%	No.	%	No.	%	No.	%
All		16 099	100.0	10 091	62.7	722	4.5	3614	22.4	912	5.7	760	4.7
Smoking status	Never	4366	27.1	3462	34.3	112	15.5	635	17.6	205	22.5	137	18.0
	Current	8520	52.9	4675	46.3	471	65.2	32	0.9	440	48.2	423	55.7
	Previous	1470	9.1	913	9.0	72	10.0	450	12.5	125	13.7	85	11.2
	Unknown	1743	10.8	1041	10.3	67	9.3	2511	69.5	142	15.6	115	15.1
Fibrosis	0/1	6468	40.2	3441	34.1	434	60.1	275	7.6	584	64.0	384	50.5
	2	464	2.9	33	0.3	19	2.6	378	10.5	93	10.2	100	13.2
	3	214	1.3	12	0.1	5	0.7	1625	45.0	65	7.1	53	7.0
	4	438	2.7	35	0.3	23	3.2	219	6.1	85	9.3	113	14.9
	Unknown	8515	52.9	6570	65.1	241	33.4	79	2.2	85	9.3	110	14.5
BMI	≤18.5 kg/m^2^	620	3.9	335	3.3	32	4.4	182	5.0	34	3.7	36	4.7
	18.5–25 kg/m^2^	8108	50.4	5108	50.6	352	48.8	1509	41.8	418	45.8	333	43.8
	>25 kg/m^2^	3350	20.8	2198	21.8	157	21.9	2080	57.6	201	22.0	148	19.5
	Unknown	4021	25.0	2450	24.3	180	24.9	539	14.9	259	28.4	243	32.0
Prior HCV	IFN + RBV	1352	80.9							713	78.2	639	84.1
Treatment	DAA + IFN	177	10.6							106	11.6	71	9.3
	DAA only	280	16.7							179	19.6	101	13.3
		Median	IQR	Median	IQR	Median	IQR	Median	IQR	Median	IQR	Median	IQR
Age, y		41	35–49	41	34–49	42	36–50	40	34–45	48	39–53	47	40–53
CD4, /mm^3^		443	288–633	437	287–618	460	291–672	410	258–610	543	369–773	550	372–754
Nadir CD4, /mm^3^		180	73–291	180	71–293	164	57–288	172	76–285	190	78–292	199	102–300
Baseline, mm/yy		01/06	01/01–05/12	01/04	01/01–07/08	03/14	03/02–02/15	02/08	01/01–11/14	01/15	10/14–07/15	12/14	09/14–05/15

Baseline was defined as the latest of January 1, 2001, enrollment to EuroSIDA, known HCV antibody status, and, for those who were HCV antibody–positive, known HCV-RNA status. Spontaneous clearers (HCV antibody–positive, HCV-RNA-negative, untreated); chronic untreated infection (HCV antibody–positive, HCV-RNA-positive, untreated); cured (HCV antibody–positive, HCV-RNA-negative, treated); treated, HCV-RNA-positive (HCV antibody–positive, HCV-RNA-positive, treated). Six hundred twenty-nine persons (45/27/197/177/183 groups 1–5, respectively) had fibrosis defined by biopsy, 1327 (102/71/537/339/278) using Fibroscan, 4807 (3089/285/961/304/168) using APRI, and 821 (285/98/410/7/21) using hyaluronic acid. All comparisons across strata had a *P* value <.0001 except prior NADM (*P* = .33), prior CVD (*P* = .0079), and HCV treatment with IFN + RBV (*P* = .0023), with DAA + IFN (*P* = .13) and DAA only (*P* = .0005).

Abbreviations: BMI, body mass index; cART, combination antiretroviral therapy; CKD, chronic kidney disease; CVD, cardiovascular disease; DAA, direct-acting antivirals; ESLD, end-stage liver disease; HCV, hepatitis C virus; IDU, intravenous drug user; IFN, interferon; IQR, interquartile range; MSM, men who have sex with men; NADM, non-AIDS-defining malignancy; RBV, ribavirin; VL, viral load.

The median follow-up (IQR) was 6.9 (3.6–13.2) years per person. During 136 084 PYFU, 1108 developed DM, for an incidence rate of 8.14/1000 PYFU (95% CI, 7.66–8.62). Of 1108 persons with DM, 354 cases were defined using HbA1c, 628 using glucose levels, 524 with a clinical diagnosis, and 521 based on use of antidiabetic medication. These figures include 225, 176, and 114 persons who were diagnosed with DM using 2, 3, or 4 methods, respectively. The crude incidence of DM stratified by HCV strata is shown in Table 2. The crude incidence of DM was highest in persons who are HCV-Ab negative (8.66/1000 PYFU; 95% CI, 8.08–9.25) and lowest in spontaneous clearers (5.62/1000 PYFU; 95% CI, 3.61–7.63). The incidence of DM in individuals who were chronically infected, successfully treated, and treated and HCV-RNA-positive was quite similar, at around 7/1000 PYFU. In univariate analyses, there was no evidence of a difference in incidence of DM across the HCV strata compared with those who were successfully treated.

**Table 2.** **Crude Incidence Rates of DM Stratified by Current HCV Strata**

		HCV-Ab Status	HCV-RNA	HCV Treatment	Events	PYFU	Rate/1000 PYFU	95% CI	Crude IRR	95% CI	*P*
Total					1108	136 084	8.14	7.66–8.62			
Group 1	Anti-HCV-negative	Negative	n/a	n/a	843	97 330	8.66	8.08–9.25	1.19	0.95–1.53	.19
Group 2	Spontaneous clearers	Positive	Negative	Untreated	30	5342	5.62	3.61–7.63	0.77	0.50–1.19	.24
Group 3	Chronically infected	Positive	Positive	Untreated	134	19 501	6.87	5.71–8.03	0.94	0.70–1.27	.69
Group 4	Successfully treated	Positive	Negative	Treated	64	8765	7.30	5.51–9.09	1.00	-	
Group 5	Treated; HCV-RNA-positive	Positive	Positive	Treated	37	5147	7.19	4.87–9.51	0.98	0.66–1.48	.94

Abbreviations: DM, diabetes mellitus; HCV, hepatitis C virus; IRR, incidence rate ratio; PYFU, person-years of follow-up.

### Association Between HCV Strata and Development of DM

**Figure 1.**
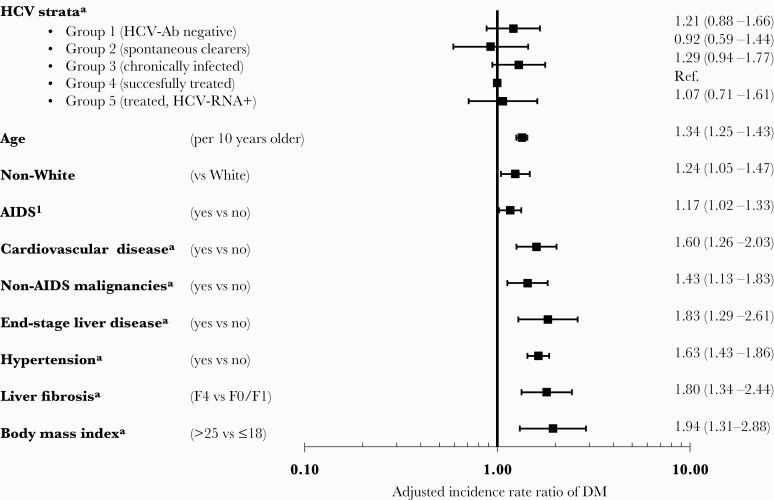
Factors associated with incidence of diabetes mellitus. Adjusted for factors shown and gender, HIV transmission risk group, nadir CD4, baseline date, glucose and HbA1C levels (as fixed values at baseline), HBsAg, cumulative exposure to zidovudine, stavudine, and didanosine, HIV viral load, CD4, smoking, chronic kidney disease as time-updated. ^a^Included as time-updated variables. DM was defined as either blood glucose >11.1 mmol/L, HbA1C >6.5%, or >48 mmol/mol, starting antidiabetic medicine, or physician-reported date of DM onset. Abbreviations: DM, diabetes mellitus; HCV, hepatitis C virus.

After adjustment, there was no difference between the HCV strata and incidence of DM, as shown in Figure 1. In a model that allowed cofactors to be updated over time (Figure 1), those who were HCV-Ab-negative (adjusted incidence rate ratio [aIRR], 1.21; 95% CI, 0.88–1.66) and chronically infected (aIRR, 1.29; 95% CI, 0.94–1.77) had the highest incidence rates of DM compared with persons who had been cured, although the increased incidence was not statistically significant, and there was no evidence of an overall difference between strata (global *P* = .33). Highly consistent results were seen in a model where all cofactors except HCV strata were fixed at baseline, with no differences between HCV strata (global *P* = .68). Other factors associated with developing DM are summarized in Figure 1 and were consistent in both multivariate models. The strongest factors associated with the development of DM were increasing age, development of cardiovascular disease (CVD), NADM, ESLD, hypertension, a high body mass index (BMI), and stage 4 liver fibrosis. There was a nonsignificantly increased incidence of DM associated with stage 2 (aIRR, 1.24; 95% CI, 0.86–1.78) or stage 3 liver fibrosis (aIRR, 1.32; 95% CI, 0.85–2.06) compared with stage 0/1 after adjustment. Our results were consistent across all our sensitivity analyses, including carrying the last HCV-RNA measurement forward for a maximum of 12 months, adjusting for starting integrase inhibitors, excluding MSM from analyses with more recent HCV infection, excluding persons with stage F3/F4 liver fibrosis at baseline, an analysis limited to post-2014, and excluding centers in EuroSIDA where the 95% CI for the center did not include the population point estimate (data not shown).

**Figure 2.**
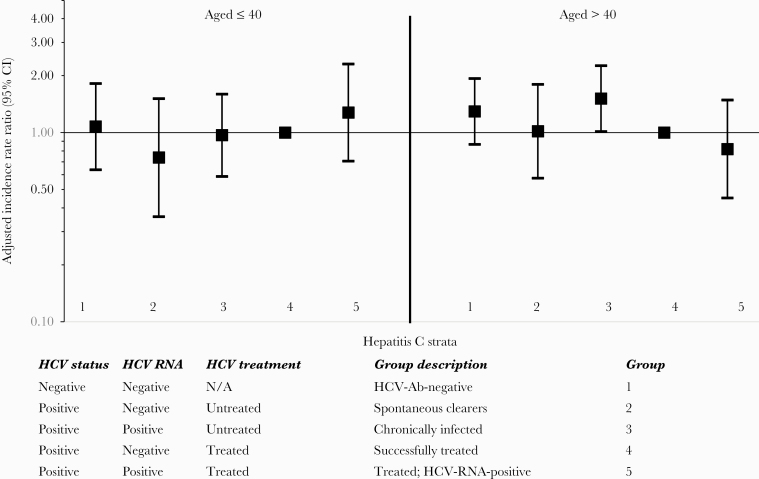
Multivariate incidence rate ratios of diabetes mellitus: Stratification by age. Adjusted for factors shown and gender, ethnic origin, HIV transmission risk group, region, nadir CD4, baseline date, glucose and HbA1C levels (as fixed values at baseline), HBsAg, cumulative exposure to zidovudine, stavudine, and didanosine, HIV viral load, CD4, smoking, AIDS, cardiovascular disease, non-AIDS-defining malignancies, end-stage liver disease, hypertension, liver fibrosis, chronic kidney disease, and body mass index as time-updated. DM was defined as either blood glucose >11.1 mmol/L, HbA1C >6.5%, or >48 mmol/mol, starting antidiabetic medicine, or physician-reported date of DM onset. Abbreviations: DM, diabetes mellitus; HCV, hepatitis C virus.

The association between HCV strata and DM differed for those aged ≤40 and those aged >40 (*P*_interaction_ = .0060). This is summarized in Figure 2. In persons aged ≤40 years, those who were chronically infected had a similar incidence of DM compared with those who had been cured (aIRR, 0.97; 95% CI, 0.59–1.60), while in persons aged >40, there was a significantly increased incidence of DM in those who were chronically infected compared with individuals who had been cured (aIRR, 1.51; 95% CI, 1.01–2.26).

### Population-Attributable Fractions for Modifiable and Nonmodifiable Risk Factors Associated With DM

**Figure 3.**
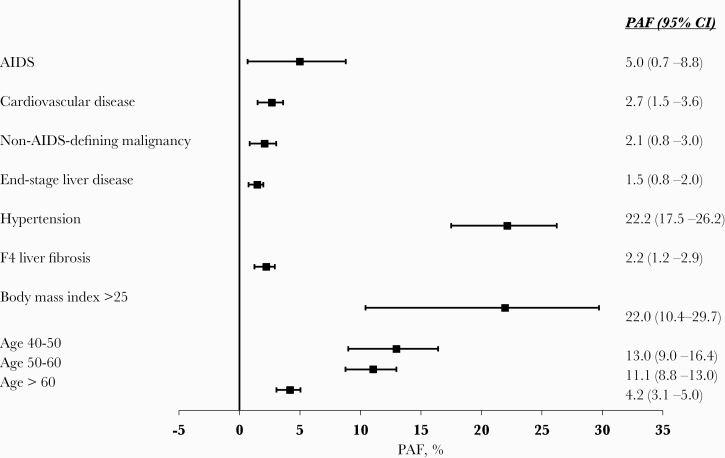
Population-attributable fractions for diabetes mellitus. Adjusted for gender, ethnic origin, HIV transmission risk group, region, nadir CD4, baseline date, glucose and HbA1C levels (as fixed values at baseline), HCV strata, HBsAg, cumulative exposure to zidovudine, stavudine, and didanosine, HIV viral load, CD4, smoking, AIDS, cardiovascular disease, non-AIDS-defining malignancies, end-stage liver disease, hypertension, liver fibrosis, chronic kidney disease, and body mass index as time-updated. DM was defined as either blood glucose >11.1 mmol/L, HbA1C >6.5%, or >48 mmol/mol, starting antidiabetic medicine, or physician-reported date of DM onset. Abbreviations: DM, diabetes mellitus; HCV, hepatitis C virus; PAF, population-attributable fraction.

The PAFs for key factors associated with DM are shown in Figure 3. The largest PAF was associated with development of hypertension, where an estimated 22.2% (95% CI, 17.5%–26.2%) of DM diagnoses could have been avoided if persons were not hypertensive. A BMI of >25 contributed 22.0% of DM cases (95% CI, 10.4%–29.7%). Comorbidities, including AIDS, ESLD, NADM, CVD, and stage F4 liver fibrosis, all contributed small but significant PAFs of ~2%–5%. Aging, a nonmodifiable risk factor, also contributed large PAFs, particularly among those aged 40–60 years. PAFs for HCV strata were not calculated, as there was no evidence of an association between HCV strata and development of DM.

Restricting the PAF analysis to those with chronic HCV infection and those treated for HCV (groups 3–5), the PAF for cirrhosis was 10.1% (95% CI, 6.2%–12.8%). The PAFs for the other factors were similar to the overall analysis except for a decreased contribution due to age >50 years (Supplementary Figure 1).

## DISCUSSION

This large cohort study is one of the largest to date of HIV/HCV-coinfected persons including >16 000 persons and 136 000 PYFU. We found no difference in the rates of DM between well-defined HCV strata including chronic and cured HCV. Traditional DM risk factors, such as age, hypertension, and obesity were much stronger predictive factors of DM than HCV status and whether someone with chronic HCV infection had been successfully treated.

We found no overall differences in the incidence of DM regarding the presence of replicating HCV infection across the HCV strata we included, and this was consistent across the different sensitivity analyses. Data from a smaller cohort study including 95 DM cases in persons with and without SVR from Berenguer et al. found a ~50% reduction in DM in those with SVR compared with those without SVR after interferon (IFN) therapy [13]. Data from the Swiss HIV Cohort Study including ~200 patients with DM found no increased incidence of DM comparing HCV-Ab-positive with HCV-Ab-negative individuals, while a comparison of HCV-Ab-positive individuals with and without SVR after IFN therapy suggested a lower incidence of DM in those with SVR, albeit with very wide confidence intervals [12]. Possible reasons for the discrepancies include differences in the populations included, that DM may develop over months or years with active HCV replication, and that a much longer follow-up period is required to observe differences, as well as differences in a study’s ability to adjust for important risk factors for DM, such as body mass index and hypertension, as we were able to in this analysis.

In a preplanned analysis, we found that the association between HCV strata and DM differed in those aged ≤40 compared with those aged >40 years. In persons aged >40 years, where the greatest burden from DM typically occurs, those with chronic untreated HCV had a significantly higher incidence of DM after adjustment compared with those who had been treated, regardless of whether the individual had cleared HCV-RNA or remained HCV-RNA-positive. Modifiable risk factors for DM, such as increasing weight and hypertension, should be closely monitored as persons age, and interventions should be targeted at those at greatest risk, such as those aged >40 years. The higher rate of DM in chronically infected persons aged >40 years may reflect a longer duration of HCV infection in older persons, which we were unable to adjust for. Our findings of lower rates of DM in individuals treated for HCV aged ≥40 years, regardless of response to treatment, could partly be explained by confounding by indication or more speculatively by improvement in lifestyle factors among those treated, described as either the “epiphany” effect [11] or the “Hawthorne” effect [20]. Further, DAA treatment in EuroSIDA began to increase most notably around 2015 [19]; before this, it is likely that the healthiest persons with lower rates of advanced fibrosis/cirrhosis and those most likely to respond to treatment were selected for interferon and ribavirin treatment with its known toxicities and limited treatment responses [21]. This channeling bias might partly explain lower rates in those treated with pegylated interferon/ribavirin than those with chronic untreated infection. Further studies focused on persons exposed to DAAs are needed to explore this association further.

Aging, hypertension, and obesity were all associated with the development of DM in our study, as previously reported and summarized by others [16, 22–24]. Smoking was not associated with development of DM, as previously reported by the D:A:D study [16]. We also found that HIV-associated comorbidities, including AIDS, non-AIDS-defining malignancies, and cardiovascular disease, were all associated with an increased incidence of DM. The association between incidence of DM and both malignancies and cardiovascular disease is likely to be explained by sharing risk factors. We have adjusted for some, such as BMI, hypertension, and aging, but there are likely to be other risk factors in common, such as physical inactivity and poor diet, that we were not able to adjust for [25]. While each of these HIV-associated comorbidities was significantly associated with DM, the PAFs were small, indicating that although they were risk factors, their underlying prevalence was low.

Whereas we did not find an association between incidence of DM and HCV infection per se, both liver cirrhosis and ESLD were associated with increased incidence of DM. These findings indicate an indirect role of HCV in increasing the risk of DM and are in agreement with findings from another recent large French cohort study of HIV/HCV-coinfected persons [7]. The liver plays a central role in the homeostasis of blood glucose levels through glycogenolysis and gluconeogenesis, although an impairment severe enough to translate into diabetes could probably only be seen in advanced or late-stage HCV-induced liver disease. The exact mechanisms through which liver cirrhosis can lead to DM are not well defined, but there is growing evidence that both insulin resistance and β-cell dysfunction contribute [26]. Even with a positive association with liver fibrosis and ESLD, the PAF was small due to the comparatively low prevalence of F4 fibrosis and ESLD among our population. In an analysis restricted to those with either chronic HCV infection or treated HCV infection, the PAF due to cirrhosis was 10%, with lifestyle factors such as increased BMI and hypertension being more important. Despite the comparatively low PAF due to cirrhosis, it remains important to diagnose and treat HCV early to reduce the risk of DM and other complications, as well as to achieve the global elimination of HCV [27]. Among the modifiable risk factors, hypertension and obesity were associated with the largest PAFs in our population. Traditional interventions used to reduce such risk factors in the general population may need modification to improve outcomes in adults with HIV in order to have a substantial impact [28]. Our results do, however, highlight the role of traditional risk factors for DM and the importance of screening and managing such risk factors.

The limitations of this study should be noted. We are not able to distinguish between type 1 and type 2 DM and used a variety of measures to define DM, reflecting heterogeneity in data collected across Europe. We focused our analyses on DM and have no data on insulin resistance, which may be associated with HCV and precede DM [29], possibly due to impaired pancreatic beta cells in HCV-positive individuals [30]. We have no information on duration of HCV infection and used the last HCV-RNA carried forward in our analysis. Where this was negative after HCV treatment we assumed SVR, consistent with our previous work [14]. As with many observational studies, our inclusion and exclusion criteria for these analyses excluded a significant proportion of persons, and there was variability in completeness of data. Using time-updated analyses and last observation carried forward includes data measured after baseline; for example, the proportion with missing data on fibrosis stage declined to 12.7% over time, of which the majority of missing data (88.8%) was among those who were anti-HCV-negative. Despite these limitations, our results were consistent across all our sensitivity analyses. We were not able to specifically address the role of DAAs in our HCV-treated groups due to limited follow-up after DAA initiation. The strength of our study is that it is one of the largest of coinfected persons reported to date, with an extensive quality assurance and data monitoring program.

In conclusion, HCV coinfection and cure of chronic HCV, mainly with DAAs, were not associated with the development of DM in this large cohort study, and there were few differences in DM across the 5 well-defined HCV strata included in this large study. The biggest modifiable risk factors of DM were hypertension and obesity, and continued efforts to manage such comorbidities should be prioritized.

## Supplementary Data

Supplementary materials are available at *Open Forum Infectious Diseases online*. Consisting of data provided by the authors to benefit the reader, the posted materials are not copyedited and are the sole responsibility of the authors, so questions or comments should be addressed to the corresponding author.

ofaa470_suppl_Supplementary_Figure_S1

## Appendix

***The EuroSIDA study group.*** The multicenter study group EuroSIDA (national coordinators in parentheses).

**Albania:** (A Harxhi), University Hospital Center of Tirana, Tirana. **Argentina:** (M Losso), M Kundro, Hospital JM Ramos Mejia, Buenos Aires. **Austria:** (B Schmied), Otto Wagner Hospital, Vienna; R Zangerle, Medical University Innsbruck, Innsbruck. **Belarus:** (I Karpov), A Vassilenko, Belarus State Medical University, Minsk, VM Mitsura, Gomel State Medical University, Gomel; D Paduto, Regional AIDS Centre, Svetlogorsk. **Belgium:** (N Clumeck), S De Wit, M Delforge, Saint-Pierre Hospital, Brussels; E Florence, Institute of Tropical Medicine, Antwerp; L Vandekerckhove, University Ziekenhuis Gent, Gent. **Bosnia-Herzegovina:** (V Hadziosmanovic), Klinicki Centar Univerziteta Sarajevo, Sarajevo. **Croatia:** (J Begovac), University Hospital of Infectious Diseases, Zagreb. **Czech Republic:** (L Machala), D Jilich, Faculty Hospital Bulovka, Prague; D Sedlacek, Charles University Hospital, Plzen. **Denmark:** G Kronborg, T Benfield, Hvidovre Hospital, Copenhagen; J Gerstoft, T Katzenstein, Rigshospitalet, Copenhagen; C Pedersen, IS Johansen, Odense University Hospital, Odense; L Ostergaard, Skejby Hospital, Aarhus, L Wiese, NF Moller, Sjællands Universitetshospital, Roskilde; L N Nielsen, Hillerod Hospital, Hillerod. **Estonia:** (K Zilmer), West-Tallinn Central Hospital, Tallinn; Jelena Smidt, Nakkusosakond Siseklinik, Kohtla-Järve. **Finland:** (I Aho), Helsinki University Hospital, Helsinki. **France:** (J-P Viard), Hôtel-Dieu, Paris; P-M Girard, Hospital Saint-Antoine, Paris; C Pradier, E Fontas, Hôpital de l’Archet, Nice; C Duvivier, Hôpital Necker-Enfants Malades, Paris. **Germany:** (J Rockstroh), Universitäts Klinik Bonn; G Behrens, Medizinische Hochschule Hannover; O Degen, University Medical Center Hamburg-Eppendorf, Infectious Diseases Unit, Hamburg; HJ Stellbrink, IPM Study Center, Hamburg; C Stefan, JW Goethe University Hospital, Frankfurt; J Bogner, Medizinische Poliklinik, Munich; G. Fätkenheuer, Universität Köln, Cologne. **Georgia:** (N Chkhartishvili) Infectious Diseases, AIDS & Clinical Immunology Research Center, Tbilisi. **Greece:** (H Sambatakou), Ippokration General Hospital, Athens; G Adamis, N Paissios, Athens General Hospital “G Gennimatas,” Athens. **Hungary:** (J Szlávik), South-Pest Hospital Centre–National Institute for Infectology and Haematology, Budapest. **Iceland:** (M Gottfredsson), Landspitali University Hospital, Reykjavik. **Ireland:** (C Kelly), St. James’s Hospital, Dublin. **Israel:** (L Tau), D Turner, M Burke, Ichilov Hospital, Tel Aviv; E Shahar, G Hassoun, Rambam Medical Center, Haifa; H Elinav, M Haouzi, Hadassah University Hospital, Jerusalem; D Elbirt, AIDS Center (Neve Or), Jerusalem. **Italy:** (A D’Arminio Monforte), Istituto Di Clinica Malattie Infettive e Tropicale, Milan; R Esposito, I Mazeu, C Mussini, Università Modena, Modena; F Mazzotta, A Gabbuti, Ospedale S Maria Annunziata, Firenze; A Lazzarin, A Castagna, N Gianotti, Ospedale San Raffaele, Milan; M Galli, A Ridolfo, Osp. L. Sacco, Milan. **Lithuania:** (V Uzdaviniene) Vilnius University Hospital Santaros Klinikos, Vilnius; R Matulionyte, Centro poliklinika, Vilnius, Vilnius University Hospital Santaros Klinikos, Vilnius. **Luxembourg:** (T Staub), R Hemmer, Centre Hospitalier, Luxembourg. **Montenegro:** (S Dragas), M Stevanovic, Clinical Center of Montenegro, Podgorica.**Netherlands:** (P Reiss), Academisch Medisch Centrum bij de Universiteit van Amsterdam, Amsterdam. **North Macedonia:** (J Trajanovska), University Clinic for Infectious Diseases & Febrile Conditions, Mother Teresa 17, Skopje. **Norway:** (DH Reikvam), A Maeland, J Bruun, Oslo University Hospital, Ullevaal. **Poland:** (B Knysz), J Gasiorowski, M Inglot, Medical University, Wroclaw; E Bakowska, Centrum Diagnostyki i Terapii AIDS, Warsaw; R Flisiak, A Grzeszczuk, Medical University, Bialystok; M Parczewski, K Maciejewska, B Aksak-Was, Medical Univesity, Szczecin; M Beniowski, E Mularska, Osrodek Diagnostyki i Terapii AIDS, Chorzow; E Jablonowska, J Kamerys, K Wojcik, Wojewodzki Szpital Specjalistyczny, Lodz; I Mozer-Lisewska, B Rozplochowski, Poznan University of Medical Sciences, Poznan. **Portugal:** (A Zagalo), Hospital Santa Maria, Lisbon; K Mansinho, Hospital de Egas Moniz, Lisbon; F Maltez, Hospital Curry Cabral, Lisbon. **Romania:** (R Radoi), C Oprea, Carol Davila University of Medicine and Pharmacy Bucharest, Victor Babes Clinical Hospital for Infectious and Tropical Diseases, Bucharest. **Russia:** A Yakovlev, Medical Academy Botkin Hospital, St Petersburg; T Trofimora, Novgorod Centre for AIDS, Novgorod, I Khromova, Centre for HIV/AIDS & and Infectious Diseases, Kaliningrad; E Kuzovatova, Nizhny Novgorod Scientific and Research Institute of Epidemiology and Microbiology named after Academician I.N. Blokhina, Nizhny Novogrod; E Borodulina, E Vdoushkina, Samara State Medical University, Samara. **Serbia:** (J Ranin), The Institute for Infectious and Tropical Diseases, Belgrade. **Slovenia:** (J Tomazic), University Clinical Centre Ljubljana, Ljubljana. **Spain:** (JM Miro), JM Miró, M. Laguno, E. Martinez, F. Garcia, JL Blanco, M. Martinez-Rebollar, J. Mallolas, P Callau, J Rojas, A Inciarta, Hospital Clinic–IDIBAPS University of Barcelona, Barcelona; S Moreno, S. del Campo, Hospital Ramon y Cajal, Madrid; B Clotet, A Jou, R Paredes, J Puig, JM Llibre, JR Santos, Infectious Diseases Unit & IrsiCaixa AIDS Research Institute, Hospital germans Trias I Pujol, Badalona; P Domingo, M Gutierrez, G Mateo, MA Sambeat, Hospital Sant Pau, Barcelona; JM Laporte, Hospital Universitario de Alava, Vitoria-Gasteiz. **Sweden:** (K Falconer), A Thalme, A Sonnerborg, Karolinska University Hospital, Stockholm; CJ Treutiger, Venhälsan-Sodersjukhuset, Stockholm; L Flamholc, Malmö University Hospital, Malmö. **Switzerland:** (A Scherrer), R Weber, University Hospital Zurich; M Cavassini, University Hospital Lausanne; A Calmy, University Hospital Geneva; H Furrer, University Hospital Bern; M Battegay, University Hospital Basel; P Schmid, Cantonal Hospital St. Gallen. **Ukraine:** A Kuznetsova, Kharkov State Medical University, Kharkov; J Mikhalik, Crimean Republican AIDS centre, Simferopol; M Sluzhynska, Lviv Regional HIV/AIDS Prevention and Control CTR, Lviv. **United Kingdom:** A Milinkovic, St. Stephen’s Clinic, Chelsea and Westminster Hospital, London; AM Johnson, E Simons, S Edwards, Mortimer Market Centre, London; A Phillips, MA Johnson, A Mocroft, Royal Free and University College Medical School, London (Royal Free Campus); C Orkin, Royal London Hospital, London; A Winston, Imperial College School of Medicine at St. Mary’s, London; A Clarke, Royal Sussex County Hospital, Brighton; C Leen, Western General Hospital, Edinburgh.

***The following centers have previously contributed data to EuroSIDA. ***Medical University, Gdansk, Poland; Infectious Diseases Hospital, Sofia, Bulgaria; Hôpital de la Croix Rousse, Lyon, France; Hôpital de la Pitié-Salpétière, Paris, France; Unité INSERM, Bordeaux, France; Hôpital Edouard Herriot, Lyon, France; Bernhard Nocht Institut für Tropenmedizin, Hamburg, Germany; 1st I.K.A Hospital of Athens, Athens, Greece; Ospedale Riuniti, Divisione Malattie Infettive, Bergamo, Italy; Ospedale di Bolzano, Divisione Malattie Infettive, Bolzano, Italy; Ospedale Cotugno, III Divisione Malattie Infettive, Napoli, Italy; Dérer Hospital, Bratislava, Slovakia Hospital Carlos III, Departamento de Enfermedades Infecciosas, Madrid, Spain; Kiev Centre for AIDS, Kiev, Ukraine Luhansk State Medical University, Luhansk, Ukraine; Odessa Region AIDS Center, Odessa, Ukraine; St Petersburg AIDS Centre, St Peterburg, Russia; Infectology Centre of Latvia, Riga, Latvia University di Roma la Sapienza, Rome, Italy; Istituto Nazionale Malattie Infettive Lazzaro Spallanzani, Rome, Italy.

***EuroSIDA Steering Committee. *Steering Committee:** I Karpov, M Losso, J Lundgren, J Rockstroh, I Aho, LD Rasmussen, V Svedhem, G Wandeler, C Pradier, N Chkhartishvili, R Matulionyte, C Oprea, JD Kowalska, J Begovac, JM Miró, G Guaraldi, R Paredes; **Chair:** G Wandeler **Co-Chair:** R Paredes; **Study lead:** A Mocroft.

***EuroSIDA staff. *Coordinating Centre Staff:** O Kirk, L Peters, A Bojesen, D Raben, EV Hansen, D Kristensen, JF Larsen, AH Fischer; **Statistical Staff:** A Mocroft, A Phillips, A Cozzi-Lepri, S Amele, A Pelchen-Matthews, A Roen.
