# Supplementary figures and images for: Influence of Hepatitis C Coinfection and Treatment on Risk of Diabetes Mellitus in HIV-Positive Persons
Source: Open Forum Infect Dis. 2020 Oct 7;7(12):ofaa470. doi: 10.1093/ofid/ofaa470 (PMC7772946; doi:10.1093/ofid/ofaa470)

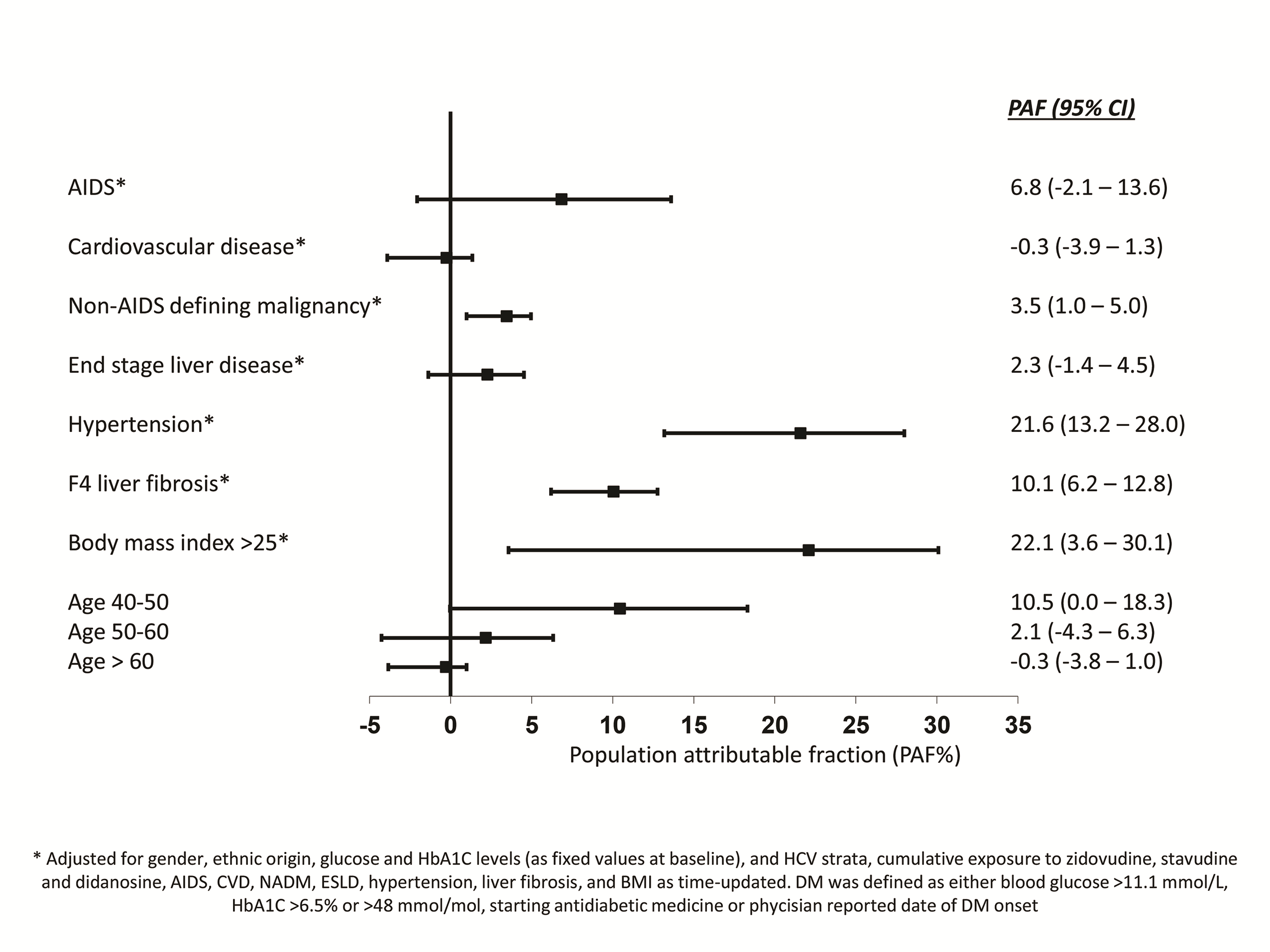

Supplement: ofaa470_suppl_Supplementary_Figure_S1 [file ofaa470_suppl_supplementary_figure_s1.png]
